# Hold on or Cut? Integrin- and MMP-Mediated Cell–Matrix Interactions in the Tumor Microenvironment

**DOI:** 10.3390/ijms22010238

**Published:** 2020-12-28

**Authors:** Stephan Niland, Johannes A. Eble

**Affiliations:** Institute of Physiological Chemistry and Pathobiochemistry, University of Münster, 48149 Münster, Germany; nilands@uni-muenster.de

**Keywords:** tumor microenvironment, extracellular matrix, integrins, matrix metalloproteinases, matrikines

## Abstract

The tumor microenvironment (TME) has become the focus of interest in cancer research and treatment. It includes the extracellular matrix (ECM) and ECM-modifying enzymes that are secreted by cancer and neighboring cells. The ECM serves both to anchor the tumor cells embedded in it and as a means of communication between the various cellular and non-cellular components of the TME. The cells of the TME modify their surrounding cancer-characteristic ECM. This in turn provides feedback to them via cellular receptors, thereby regulating, together with cytokines and exosomes, differentiation processes as well as tumor progression and spread. Matrix remodeling is accomplished by altering the repertoire of ECM components and by biophysical changes in stiffness and tension caused by ECM-crosslinking and ECM-degrading enzymes, in particular matrix metalloproteinases (MMPs). These can degrade ECM barriers or, by partial proteolysis, release soluble ECM fragments called matrikines, which influence cells inside and outside the TME. This review examines the changes in the ECM of the TME and the interaction between cells and the ECM, with a particular focus on MMPs.

## 1. Introduction

**Figure 1:**
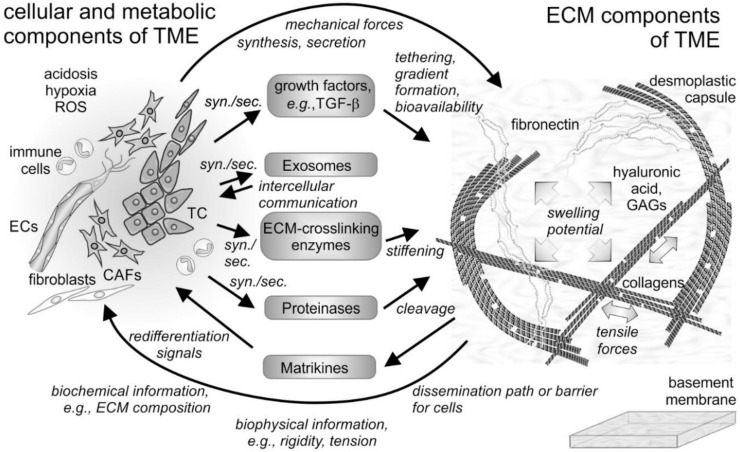
Cellular and non-cellular components of the tumor microenvironment (TME) and their interplay. In addition to the tumor cells (TCs), fibroblasts and their derivatives, the cancer-associated fibroblasts (CAFs), as well as ingrowing endothelial cells (ECs) and infiltrating immune cells are the cellular components of the tumor microenvironment (TME). The cells synthesize and secrete (syn./sec.) not only the extracellular matrix (ECM) components, but also growth factors, exosomes, and ECM-modifying enzymes, such as proteinases. Both cells and their secretion products interact with the fibrillar and non-fibrillar components of the ECM. Hyaluronic acid and glycosaminoglycan (GAG)-chains of proteoglycans increase the swelling potential of the interstitial space, which is counterbalanced by the tensile force-bearing fibrils of collagens, elastin and fibronectin. Modified by tethered growth factors, by crosslinking and cleaving enzymes, and by contractile forces, the ECM and its fragments with cytokine-like functions (matrikines) influence the cells within the TME in various ways. ROS, reactive oxygen species.

The tumor microenvironment (TME) describes the conditions within and in the vicinity of a solid tumor mass. It is shaped in an orchestrated manner by the oncogenically transformed cells and their neighboring tissue cells. It comprises cellular and noncellular constituents of macromolecular size and smaller molecules, as well as several biophysical parameters, such as pH, redox status [1], and mechanical tension within the tissue [2,3] (Figure 1). Among the small molecules, aberrant concentrations of redox potential-determining compounds, such as glutathione and reactive oxygen species (ROS) as well as extracellular ATP, characterize the TME [1].

Fibrillar and non-fibrillar proteins and rather amorphous proteoglycans together form the insoluble scaffold of the extracellular matrix [4,5] (Figure 1). The TME is also unique in its composition of soluble extracellular proteins, such as cytokines [6,7] and enzymes. Among the latter, extracellular matrix (ECM)-degrading matrix metalloproteinases (MMPs) and ECM-crosslinking lysyl-oxidases and transglutaminases characteristically contribute to the plasticity and distinction of the TME [8,9,10,11]. They are actively produced and remodeled by cells of the tumor mass and constitute the surroundings, which influence cancer cells and their neighboring cells in a tumor-supportive manner. This review will shed light on the ECM of the TME and will take into account its TME-characteristic remodeling with a special emphasis on the MMPs. Moreover, it will summarize the current knowledge on the interactions of TME-embedded cells, both cancer and resident cells, with the ECM and the mutual effects on each other in maintaining tumor-supportive surroundings and in fostering metastasis.

## 2. The Extracellular Matrix as a Key Component of the TME

The palpation of, e.g., the mammary gland [3], is a simple procedure to detect volume-demanding and stiffer tumor tissue [2,12]. Biophysical differences between normal tissue and tumor mass are caused by cell growth and the increased deposition of ECM components, known as desmoplasia, which is typically observed in healing wounds and fibrosis [13].

Most of the mass of solid tumors consists of ECM [14]. Having developed from collagen-rich stromal tissue, the TME is rich in collagens, especially if the tumor mass induces a desmoplastic reaction [13,15]. Collagens as the most abundant proteins of the human body crucially contribute to the scaffolding function of the ECM. The almost 30 members of the collagen family share several characteristics: (i) Their three chains consist of the repetitive Gly–X–Y amino acid sequence with X and Y being different amino acids, most frequently proline and hydroxyproline; (ii) They form a characteristic, staggered triple helix with the glycine residues of all triplet sequences in its center; (iii) They self-assemble into supramolecular structures, in which several triple-helical collagen molecules associate forming fibrils, networks, and other highly ordered aggregates [5,14,16]. Fibrils of type I collagen, together with collagen types III and V, bear the tensile forces within normal stromal tissue and in the TME of the tumor mass. They are preferentially deposited in desmoplastic environments, where the resident stromal cells are induced by tumor cells to produce and deposit collagen type I to form the stiff TME or a capsule surrounding the tumor mass [13,15]. Another collagen isoform, type IV collagen, along with collagens XV and XVIII, forms a network-like suprastructure, which is typical of basement membranes (BMs), the specialized sheet-like ECM that separates stromal tissue from other tissues. As it confines cells to their respective tissue type, its breaching by malignant cells is a hallmark of cancer [17].

**Figure 2:**
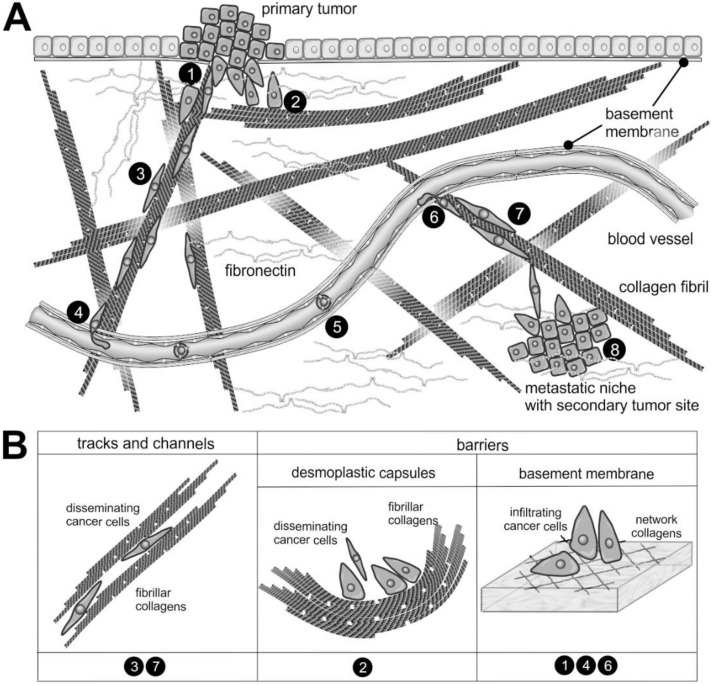
The supramolecular structure of collagen as a substrate or impediment of cancer cell dissemination: (**A**) The metastatic cascade of malignant carcinoma cells includes the penetration of the epithelial basement membrane (❶); Solid tumors within the stroma tissue are often surrounded by a desmoplastic collagen capsule, which impedes cancer cell migration (❷); Through the interstitial stroma, cancer cells utilize collagen-rich fibrils to quickly reach a nearby blood vessel (❸); There, they intravasate through the endothelial basement membrane (❹); When blood-borne, they float with the blood stream, mostly sheltered by platelets (❺); To extravasate, they attach to the vessel wall of a distant organ and again breach the endothelial basement membrane (❻); Moving quickly along collagen-rich fibrils (❼), they reach the metastatic niche (❽), where they grow into a metastasis. (**B**) The different dissemination-supporting and impeding functions of the collagen suprastructures are highlighted for the different steps of the metastatic cascade. The cancer cells experience, particularly by integrins, the ECM as dissemination-supporting tracks and channels, especially if migration and fibrils point into the same direction and if the fibril network is not too dense. In contrast, the dense array of collagen fibrils in desmoplastic capsules and of network-forming collagens within the basement membrane impedes cancer cell progression and requires the use matrix-metalloproteinases. Moreover, the orientation of collagen fibrils within the desmoplastic capsule is mostly perpendicular to the direction of dissemination [28].

Spanning the interstitial stroma, collagen fibrils provide an ideal path for cell migration and promote cancer cell dissemination along these fibrils (Figure 2). In contrast, the meshwork of stromal collagen fibrils and the desmoplastic capsule around the tumor mass like the type IV collagen network of basement membranes are extremely dense and impede tumor cell infiltration [18,19]. These ECM barriers are overcome by the cancer cells or their accompanying CAFs by the cleavage of collagen with particular collagenases [20].

In addition to the network-forming collagens, laminins, which form a family of about 20 members, are typical constituents of basement membranes [21,22,23]. Their N termini and the C terminus formed by the globular G domain of the laminin α chain protrude from an α-helical coiled coil [21]. Although laminins are normally exclusively found in BMs, some types of laminins, such as laminin-332, also occur ectopically within the TME [24], but their role in the TME has not yet been fully deciphered.

Collagen fibrils are often found together with elastin and fibulin containing elastic fibrils [25], which, due to their reversible elasticity, allow the resilience of the ECM. Interestingly, most of the body’s elastin is formed pre- and early postnatally but hardly in the adult body [26]. However, some cancer entities stimulate and reinitiate elastin production and deposition, which is known as elastosis [25]. Similarly noteworthy, elastin degradation peptides (EDPs) are released in the TME, which stimulate tumor cell growth and progression via different receptors [27] (Figure 2).

Fibronectin is another scaffold-forming glycoprotein found in BMs as well as in the ECM of the TME [29,30], with distinct splice variants being produced in the TME [31]. Fibronectin consists of two disulfide-linked protein chains with a characteristic modular character of fibronectin repeats of types I, II, and III with about 30, 60, and 90 amino acids, respectively. The type I and II repeats allow the formation of disulfide-crosslinked supramolecular fibronectin networks in the tissue stroma [30]. Fibronectin isoforms with the extra domains ED-A and ED-B are normally expressed under the control of mainly TGF-β during embryonic development and wound repair but also in the hypoxic TME [32,33]. Thus, ED-A- and/or ED-B- fibronectin are employed as a marker to image tumor nodes [34]. Other glycoproteins marking a tumor-modified stroma are tenascins-C and W [35,36,37]. Like the other two members of the tenascin family [38], they are disulfide-crosslinked homotrimers, each consisting of three chains forming an α-helical coiled coil. With their ECM-typical EGF-like and type III fibronectin domains, and a C-terminal fibrinogen globe module, they perform various functions in wound healing and tumor progression [36]. These tenascin isoforms or fragments thereof can also be released from the primary tumor into the blood circulation and precondition distant sites as premetastatic niches [39]. Thus, the blood levels of tenascins are a diagnostic tumor marker [40]. Their abundance in the TME is tested for diagnostic imaging and therapeutic exploitation [41]. Tenascins and other pericellular ECM proteins, such as periostin [42], galectins [43], small integrin-binding ligand N-linked glycoproteins (SIBLINGs), secreted protein acidic and rich in cysteine (SPARC), thrombospondin, angiopoietin-like proteins, certain proteoglycans, and CCN family members are referred to as matricellular proteins [44]. Rather than performing scaffolding functions, they modulate the supramolecular architecture of the collagen and fibronectin network [42] and they regulate cellular behavior within the TME [37]. For example, CCNs regulate the proliferation and migration of ECM scaffold-embedded cells [44,45].

Functionally similarly versatile are glycosaminoglycans (GAGs), and the protein- and sulfate-free hyaluronic acid (HA) made up of N-acetylglucosamine and glucuronic acid [46,47], as well as sulfated GAG-chain containing proteoglycans, such as heparan sulfate and keratan sulfate, and galactosamine-containing chondroitin sulfate and dermatan sulfate [4,5,48,49]. According to their location, proteoglycans are divided into extracellular, membrane-bound, and intracellular proteoglycans. The extracellular group includes the hyaluronic acid-binding hyalectans (e.g., aggrecan, versican with four different splice variants, neurocan, and brevican), the small leucine-rich proteoglycans (SLRPs, e.g., decorin, biglycan, fibromodulin, and lumican), and the BM-located pericellular proteoglycans (e.g., perlecan, agrin, and collagen XVIII). Perlecan, agrin, and the network-forming type VIII collagen bear heparan sulfate GAG chains [48], while the hyalectans and SLRPs are decorated with different numbers of covalently attached chondroitin sulfate, sometimes in combination with additional dermatan sulfate chains (decorin) or keratan sulfate chains (aggrecan, fibromodulin, lumican) [49]. The extracellular proteoglycans non-covalently link up with the scaffold of fibril-forming ECM components in different ways. Type IX collagen decorates the surface of type II collagen fibrils, while type XVIII collagen forms the BM-typical chicken wire network together with type IV collagen. Although alloyed into collagen fibrils, they are also proteoglycans due to their chondroitin sulfate and heparan sulfate GAG-chains, respectively [4,5]. Alternatively, several proteoglycans, such as decorin and other SLRPs, interact via their protein cores with type I collagen-containing fibrils and thus control the suprastructure of the ECM scaffold [46]. Moreover, specific type III repeats of fibronectin, certain G-domains of laminin α-chains, tenascins and thrombospondins harbor heparan sulfate binding sites, thereby allowing protein-carbohydrate binding interactions to connect the “amorphous” proteoglycans with higher suprastructure-forming ECM components [5]. Likewise, the hyalectans have a carbohydrate-binding domain, which allows their binding to hyaluronic acid.

Proteoglycans with their GAG chains also specifically tether growth factors and assist in presenting them to the respective cellular receptors. Such tethering of growth factors and cytokines stabilizes the formation of spatial gradients, which are indispensable for normal development and also contribute to pathologic processes. For example, in tumor angiogenesis, endothelial cells (ECs) follow a VEGF gradient that is stabilized by tethering to heparan sulfate GAG chains [47,48].

## 3. Fibrillar and Non-Fibrillar ECM Components Orchestrate the Biophysical and Biochemical Properties of the TME

Even if the cellular origin of the ECM and its impact on the cells of the TME were neglected, the different ECM components alone would interplay in a manner that would already explain some of typical features of the TME, e.g., its increased stiffness and tension [2]. Due to its high charge density, GAGs, either as hyaluronic acid or as carbohydrate conjugates of proteoglycans, absorb tremendous amounts of water, causing their immense swelling capacity [50]. However, they do not reach their equilibrium swelling volume due to the limited space of the ECM scaffold and due to the tight connections between the ECM network with interwoven proteoglycans. Thus, the tissue pressure is counteracted by tensile forces, which are taken up by the fibrillar ECM components. This tensed force equilibrium shapes the interstitial stroma tissue and contributes to the cushion function of the ECM [14,15]. The enhanced deposition of hyaluronic acid and proteoglycans in the tumor mass, e.g., in pancreatic duct carcinomas, is accompanied by a desmoplastic increase in collagens [13,51] resulting in increased tissue rigidity and intra-tissue tension [2]. Embedded fibroblasts exert mechanical forces on the ECM scaffold and thus increase its stiffness and tension [52]. At the same time, they also sense changes in the biophysical properties of the TME and adapt their behavior (see below). The increased interstitial pressure of the TME impedes the access of anti-cancer drugs to the tumor mass [1,2,53].

The increased tensile forces on the fibrillar ECM proteins also regulate the bioavailability of growth factors deposited on them, such as TGF-β, one of the key growth factors of the TME [54,55]. Its preform, the latency-associated peptide (LAP) consisting of two non-covalently associated chains, is released after intracellular furin cleavage and tethered by fibrillin to elastic fibrils. When mechanical forces stretch the fibrillin, LAP detaches and becomes accessible to extracellular proteases that release active TGF-β, which then binds to its respective receptors and triggers de- and re-differentiation of cells within the TME [56].

## 4. The “Give and Take” between the ECM and Cells within the TME

The cellular players within the TME are as complex as the ECM. Formerly, tumor research focused on cancer cells, their oncogenic transformation, metabolic reprogramming, proliferation, dissemination and metastasis. This has provided important knowledge on cancer cells, their diagnostic detection and therapeutic targeting. However, recent decades have also highlighted the important role of resident and infiltrating cells of the TME, which under the influence of the nearby cancer cells, change their properties and metabolism in a tumor-supportive manner. This has broadened the perspective of cancer research and created a more holistic picture of cancer cells and their neighboring cells, among them resident fibroblasts, ingrowing ECs and infiltrating immune cells [57]. Especially the fibroblasts have become important in the context of the TME, as they are the major source of ECM components [58,59]. The ingrowth of ECs and the infiltration of immune cells also significantly contribute to the TME and are in turn influenced by the TME [60], which, however, is beyond the scope of this article and examined elsewhere [61,62,63].

Under the influence of cancer cells, tissue-resident stromal fibroblasts differentiate into cancer-associated fibroblasts (CAFs) [60,64,65]. However, CAFs are a heterogeneous group of cells and may originate from other cell types as well [52,65,66]. Due to their importance in tumor progression, CAFs are now considered as a promising target in cancer treatment [65,67]. Several clinical trials directed against CAF-marker proteins have also been launched to overcome drug resistance in treatment of cancer [68]

Cancer cells secrete growth factors, such as TGF-β [55], that promote the differentiation CAFs from resident fibroblasts [69]. Similar to fibrosis-associated fibroblasts in wound healing, CAFs synthesize, secrete, and deposit TME-typical ECM components, and contract the tissue with their α-smooth muscle actin (αSMA)-reinforced cytoskeleton [70]. The more rigid the ECM of the TME, the more its tension fosters CAF differentiation with YAP/TAZ acting as a sensor for structural and mechanical features of the ECM [71].

The repertoire of cellular adhesion molecules (CAMs) is in turn modulated by the altered ECM of the TME. Among them are hyaluronic acid receptors, membrane-bound proteoglycans, and integrins. They anchor the cells to the ECM scaffold allowing the transmission of cellular forces onto ECM components, and serve as signal relays transmitting cues of the ECM into the cells and vice versa. However, unlike growth factor receptors, most of the CAMs lack kinase domains and signal via associating proteins.

The hyaluronic acid receptors CD44 and RHAMM are closely linked to cancer progression and dissemination. With its known signaling mechanisms [72], CD44 has dual functions in the TME that can both support and attenuate tumor progression, depending on the differential expression, processing and fragmentation of its ligand, hyaluronic acid, in the course of tumor growth [73]. Newly synthesized high molecular weight hyaluronic acid (HMW-HA) is bound by CD44, while at a later disseminating stage of tumor progression, HMW-HA is fragmented by hyaluronidases and the resulting low molecular weight (LMW)-HA fragments. While LMW-HAs do also bind to CD44 and thereby exert important regulatory roles in the TME, as they inhibit CD44 clustering and have an antagonistic effect on HMW-HA activity, LMW-HAs are preferentially recognized by RHAMM [74,75], which is upregulated in disseminating cancer cells and alters the HA signaling of CD44 [74,75].

Furthermore, membrane-bound proteoglycans, such as syndecans, glypicans, and betaglycan, influence interactions between cancer cells and the TME [50]. Notably, the expression of the transmembrane proteoglycan NG2 (CSPG4) is altered on cancer cells and CAFs and may serve as a CAF-marker [49]. The mode of action of membrane-bound proteoglycans is diverse. In addition to tethering growth factors, such as TGF-β, VEGF, and hepatocyte growth factor (HGF), they act as co-receptors too [76,77,78]. Betaglycan is also known as TGF-β type III receptor [5]. Syndecans serve as co-signaling receptors for integrins and modulate their activity [79,80,81]. Soluble SLRPs also interact through their protein core with the extracellular domains of growth factor receptors and integrins, thereby regulating their extracellular ligand binding. For example, decorin, affects the binding of α2β1 integrin to collagen [82], and also the receptors for VEGF, HGF, and epidermal growth factor (EGF), as well as the immunomodulatory Toll-like receptors (TLR)-2 and -4 [76,83,84], the latter preferentially binding biglycan [84]. A similar receptor modulation appears to be effective in the fragmentation of the pericellular proteoglycan perlecan, whose N-terminal fragment endorepellin specifically binds to integrin α2β1 [85,86].

The integrins are undoubtedly the most extensive family among the CAMs in almost all multicellular organisms [87,88,89]. There are 24 different heterodimeric integrins, each consisting of one of 18 α-subunits and 8 β-subunits. The β1-containing integrins α1β1, α2β1, α10β1, and α11β1 recognize collagens, and the β3-containing integrins α3β1, α6β1, α6β4, and α7β1are laminin receptors, whereas fibronectin can be bound by the integrins α5β1, αVβ1, αVβ3, and αVβ6 [87,88]. Like other CAMs, they mechanically anchor the cells in the ECM and also transduce signals between cells and their environment. For this purpose, integrins are associated with the actin cytoskeleton and its motor proteins and form the transmembrane connectors to the ECM scaffold, on which they exert tensile [90]. With their integrins, CAFs, contribute significantly to the interstitial tissue pressure typical of the TME [91], whereby integrin αvβ3 in this context can apparently replace collagen binding β1 integrins [92]. In integrin signaling, significant conformational changes, and clustering into a supramolecular array on the cell surface, as well as the recruitment of several adapter and signal molecules such as kinases and G-proteins are involved [93,94,95,96,97]. This results in the formation of a new cell organelle, the adhesome, a complex supramolecular network of proteins with a highly ordered and hierarchical structure [98,99]. During the oncogenic transformation, the ECM ligand binding activity is regulated by changing various parameters, such as extracellular and intracellular ligands, e.g., the proteoglycan decorin or intracellular kindlins, as well as divalent cations and protons, redox-active compounds, and mechanical forces [82,100,101,102,103,104,105]. Therefore, integrin expression and surface abundance on cancer cells can serve as tumor (suppressor) markers, although this may vary between tumor entities [91,106,107,108]. For example, the expression of α3β1 and α11β1 integrins changes during CAF differentiation, whereupon CAFs interact via these integrins with ectopically expressed laminin-332 and desmoplastically abundant collagen, respectively, and thus support cancer cells [24,109]. Moreover, integrin expression and function is regulated by the TME characteristic TGF-β, and vice versa [110].

Although many studies have been undertaken to define whether certain ECM-proteins, such as fibronectin, and their receptors promote or attenuate tumor progression and spreading, a comprehensive picture of the role of all ECM proteins within the TME has not been fully drawn yet [30]. This is due to the fact, that the ECM of the TME can be different for certain tumor entities [111] and for different stages of tumor progression [30]. Likewise, the role of the ECM receptors is diverse and not entirely defined for each cell type within the TME at any stage of tumor progression and dissemination [72,75,78,112]. However, the ECM contacts of cells in the TME affect inter alia the secretion and deposition, as well as the remodeling of ECM components. Thus, the ECM–CAM axis is a way of signaling between the TME and its cells comparable to paths of paracrine signaling, via growth factors, cytokines and exosomes [69,70,113,114,115], which are all interdependent [116] and additionally integrate other features of the TME, such as the lactacidosis, hypoxia, and altered levels of ROS [117,118,119].

The remodeling of the ECM within the TME includes biochemical changes of its composition as well as the biophysical alteration of tissue rigidity and tension [2,3,12]. In addition, cells of the TME also secrete ECM-modifying enzymes, such as covalently crosslinking enzymes that stabilize ECM fibrils and stiffen the TME [15], but also ECM scaffold-cleaving proteases, such as MMPs [31,70,120]. Members of the lysyl-oxidases and of the transglutaminases, especially the isoforms LOXL2 and transglutaminase 2, respectively, connect the ECM proteins via uncleavable linkages and isopeptide bonds [10,121,122]. Thus, they determine not only the TME of the primary tumor site, but also determine the premetastatic niche by modifying the ECM scaffold of distant tissues [3,117,118,119,123]. Proteases secreted into the TME are also a means of communication between cells and the ECM [124]. They specifically cleave ECM components to release soluble ECM fragments with signaling functions for the cells, called matrikines [125]. Furthermore, they degrade proteins of the ECM, thereby weakening its scaffold structure, or locally open the ECM barrier for disseminating cancer cells accompanied by CAFs [19,124]. The expression and function of ADAMs (a disintegrin and a metalloproteinase) in the latter has recently been reviewed [126]. GAG-degrading hydrolases such as hyaluronidases are also involved in ECM remodeling [127]. A dysfunctional imbalance in ECM synthesis and degradation leads to desmoplasia and fibrosis. Among the matrix-degrading enzymes, MMPs play a central role in the entire tumor microenvironment [128].

## 5. Cancer Progression is Dependent on the Proteolytic Action of MMPs on the ECM of the TME

### 5.1. The Metastatic Cascade

In such a tumor microenvironment with mutually influencing ECM and cells located therein, the metastatic cascade begins. This comprises several steps by which cancer cells disseminate from the primary tumor, spread throughout the body, and metastasize into distant organs (Figure 2). First, a tumor cell leaves the primary site and enters the bloodstream via the lymph or, with the help of proteolytic enzymes, breaches the vascular wall and thus enters the bloodstream directly. On its route, it breaks through various barriers, such as the EC layer during intra- and extravasation, BMs and other scaffolds of extracellular matrix (ECM) molecules [5].

While developing epithelial neoplasms remain in their original location, the main characteristic of malignancy is that cancer cells destroy the BM that separates the epithelium from the adjacent connective tissue [17]. In particular, the dense network of the BM is impermeable to cells with the exception of immune cells and malignant tumor cells. Such cells secrete numerous different proteases [18,129,130,131]. The synthesis and secretion of MMPs by cancer cells depends on various TME factors, such as special growth factors and tumor-specific variants of fibronectin [132]. Among these proteases, the 28-member family of Zn^2+^-dependent matrix metalloproteinases (MMPs) plays an important role, and MMPs are therefore of prognostic importance [133,134,135,136,137]. Collagens are particularly resistant to proteolytic attack due to their triple-helicity but can be cleaved by collagenases outperforming gelatinases that can cleave only single strands of denatured collagen [5]. The main interest from the beginning was therefore in the two collagenases MMP-1 and MMP-14 (MT1-MMP), the latter of which is membrane bound, and in the gelatinases MMP-9 and MMP-2 [135,138,139,140,141,142,143,144,145]. Collagenolytic, MMPs partially untwist the collagen triple helix with their hemopexin domain and then cleave the now accessible chains with their catalytic domain [146]. The collagenolytic cleavage destabilizes the comparatively proteolysis-resistant triple helix of the resulting fragments, which partially unwind and thereby become substrates for gelatinases MMP-2 and -9 [20,147,148]. MMP-14 also activates MMP-2 in a complex binding and cleavage mechanism [20,149] with collagen-binding β1 integrins, especially α2β1, which bring collagen to MMP-14 for proteolytic cleavage.

### 5.2. Structural and Functional Diversity of Matrix Metalloproteinases

**Figure 3:**
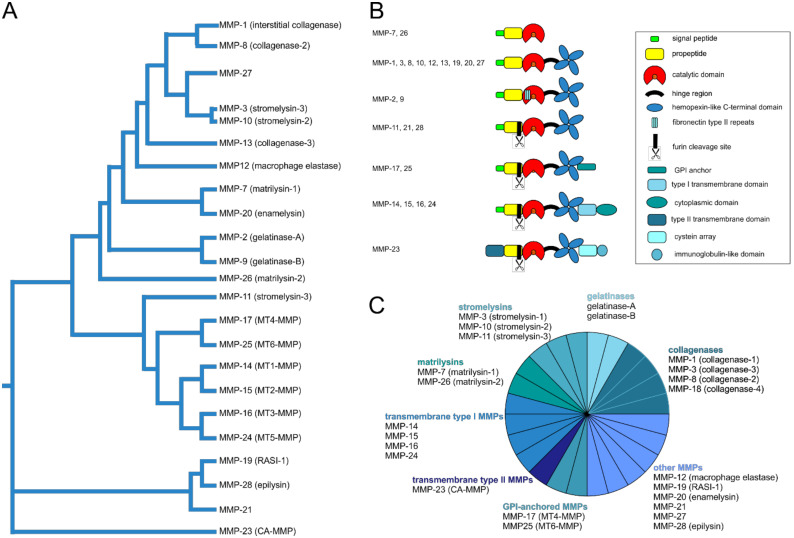
Phylogenetic and functional relationship of human matrix metalloproteinases (MMPs). In addition to the numbering of the MMPs, which corresponds to the order of their discovery, the MMPs can be ordered according to their sequence similarity [154,155] **(A)**. Regarding their domain organization **(B)** and substrate specificity they can be assigned to different groups within the MMP family (**C**): soluble collagenases, gelatinases, stromelysins, matrilysins, membrane-anchored transmembrane type I and type II as well as glycosylphosphatidylinositol (GPI)-anchored MMPs, and other MMPs [152]. All MMPs except MMP-23 have a propeptide with an N-terminal signal sequence. To activate an MMP, this propeptide has to be cleaved off in order to make a zinc ion in the active site of the catalytic domain, which also holds calcium ions, accessible through a cysteine switch. CA-MMP, cysteine array matrix metalloproteinase; RASI-1, rheumatoid arthritis synovium inflamed-1.

Matrix metalloproteinases (MMPs) are of crucial importance for invasive cancer cells to break ECM barriers (Figure 3). Within the metzincin superfamily, which also includes astacins, reprolysins, meprins and ADAMs, MMPs form a family of zinc- and calcium-dependent endopeptidases that can cleave all BM and ECM molecules [150]. Of the 28 MMPs identified in vertebrates, 24 are found in humans, including two MMP-23 isoforms encoded at different loci (MMP-23A and MMP-23B) [151,152,153]. The MMP family members can be divided according to their sequence similarity, domain organization and substrate specificity, into (i) collagenases, (ii) gelatinases, (iii) stromelysins, (iv) matrilysins, (v) transmembrane type I, (vi) transmembrane type II, (vii) glycosylphosphatidylinositol-anchored (GPI-anchored), and (viii) other MMPs (Figure 3) [9,150].

In addition to their structural and functional similarities, the individual MMPs are characterized by their distinct domain structure [137,152]: equipped with an N-terminal signal and a propeptide, MMPs have a catalytic metalloproteinase domain that is linked by a hinge region to a hemopexin domain. This central catalytic domain contains a catalytically active zinc ion chelated by three histidine residues in the binding motif HEXXHXXGXXH within the active center of the catalytic domain. Another zinc ion and two to three calcium ions within the catalytic domain have structural roles and account for substrate specificity. In addition to these characteristics common to all MMPs, MMPs have individual structural features. While gelatinases are characterized by three type II fibronectin repeats in their catalytic domain, matrilysins have neither a hinge region nor a hemopexin domain. Furin-dependent MMPs have a furin-like pro-protein convertase recognition sequence at the C-terminus of their propeptide. Unlike the soluble MMPs, membrane-bound MMPs of the membrane type (MT-MMPs) have an additional transmembrane domain and a cytosolic domain that consists of the case of MMP-23 of a cysteine-rich region and an immunoglobulin-like domain, or they are attached to the membrane with a glycosylphosphatidylinositol (GPI) anchor. The transmembrane domain is usually located towards the C-terminus with the only exception of again MMP-23, which instead of a propeptide, has an N-terminal type II transmembrane domain, which contains the sequence ALCLLPA instead of the consensus motif PRCGXPD that keeps MMP-23 it in its latent proMMP form.

proMMPs are activated to MMPs via limited proteolysis by trypsin, other MMPs, plasmin or furin-like convertases. By splitting off the propeptide, the latent proMMP is converted into its active form by means of a cysteine switch, as the removal of the cysteine sulfhydryl group in the sequence PRCGXPD, or PRCGVTD for MMP-28, of the propeptide, opens the catalytic site for the substrate molecule. Alternatively, some MMPs are also allosterically activated by a substrate molecule binding to a so-called MMP exosite outside the catalytic domain, or by chemicals, such as ROS, that affect the binding of the catalytic zinc ion to the cysteine thiol group of the cysteine switch [156].

### 5.3. MMP-14 Decisively Controls Tumor Progression

Only a few MMPs (MMP-1, -8, -13, and -14) are capable of cleaving triple helical collagen. Membrane-bound MT-MMPs with a C-terminal transmembrane domain can shed membrane proteins and cleave pericellular ECM components. Among them, MMP-14 is the only collagenolytic membrane-bound MMP [141]. It is expressed by various cell types, e.g., ECs and adipocytes [157], and moreover, it is of key importance on cancer cells [158,159,160]. Its expression correlates with their metastatic potential and is an important prognostic marker, e.g., in breast cancer [141,143,161]. In addition, CAFs express MMP-14 and contribute to invasion and metastasis in a murine breast cancer model [162]. Consequently, MMP-14 decisively controls collagen turnover and the breaking of ECM barriers in tumor progression. Its deficiency cannot be compensated for by other MMPs, neither in physiological developmental processes nor in cancer progression. In mice, MMP-14 ablation results in delayed ossification, decreased angiogenesis, severe fibrosis, and early lethality, and MMP-14 silencing with siRNA effectively reduces cancer cell invasion as does the proteolytic removal of the MMP-14 ectodomain from its transmembrane domain [159,160,163]. Conversely, the overexpression of MMP-14 promotes the invasiveness of cancer cells in a collagen-rich environment [158]. The MMP-14-cleavable substrates comprise fibril-forming collagen types I, II and III and other ECM proteins, in particular BM laminins and laminin-332 ectopically expressed in the tumor stroma [141]. In addition, MMP-14 can release membrane-bound cell and matrix receptors such as E-cadherin [164], syndecan-1, and the hyaluronan-receptor CD44 [165] from the cell surface. The outstanding importance of MMP-14 is further underpinned by its ability to activate the zymogen forms of the soluble gelatinases MMP-2 and -9 as well as the collagenolytic MMP-13.

Another yet non-proteolytic activity of MMP-14 that is of particular interest in the context of hypoxic TME is the ability of MMP-14 to activate hypoxia-inducible factors (HIFs) via Munc18-1-interacting protein 3 (Mint3) and factor inhibiting HIF-1 (FIH-1), thus promoting the expression of HIF target genes and the Warburg effect ([166] and references therein).

### 5.4. Regulation of MMP Expression

MMPs play a central role in the matrix-degrading enzyme activity that is associated with cancer metastasis and secondary tumor development. In most cases, there are no underlying gene amplifications or activating mutations. Instead, the MMP activity is dysregulated [167,168].

**Figure 4:**
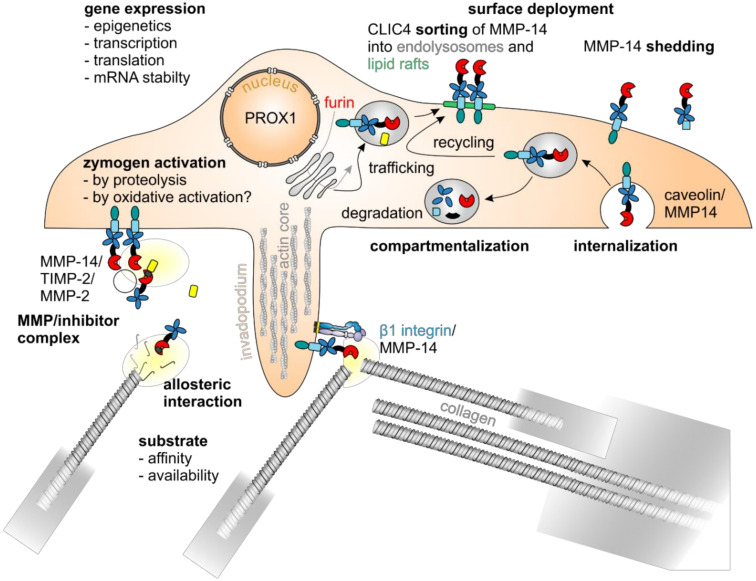
MMP-14 is regulated by various mechanisms. The enzymatic activity of MMP-14 is regulated by various mechanisms: at the level of gene expression, there are epigenetic [172], transcriptional, and posttranscriptional regulatory mechanisms, such as the cotranslational cleavage of its signal sequence at the endoplasmatic reticulum, the furin-mediated removal of its self-inhibitory prodomain within the Golgi compartment [174], the O-glycosylation of protease-sensitive linker regions, and the phosphorylation as well as palmitoylation of its cytoplasmic domain [175,176], with the transcription factor PROX1 playing an important role [157]. Zymogen activation [156], compartmentalization, surface deployment and internalization [177,178], sorting into lipid rafts [179], homodimerization and interaction with other proteins, such as TIMPs [180], integrin β1 [181], and substrates, as well as shedding [182,183] are closely interlinked.

To prevent accidental proteolysis, the gene expression of MMPs is strictly controlled at intracellular and extracellular levels by cell–matrix and intercellular interactions and the input of various growth factors, glucocorticoids, cytokines, retinoic acid, interleukins and eicosanoids [169,170]. All of these factors induce the expression of MMPs via, for example, NFκB, MAPK and JAK/STAT signaling ([171] and references therein). There is some degree of co-regulation, as several MMP promoters share structural features [169]. Notably, the expression of MMPs-2, -14, and -28 is less responsive than that of others to cytokines and growth factors [9]. Nonetheless, MMP-14 in particular, with its diverse functions, must be precisely regulated (Figure 4). Accordingly, MMP-14 is subject to epigenetic control by histone modification, chromatin remodeling and DNA methylation-sensitive transcription factors such as SP1 [169,172,173]. The methylation status of MMP-14 and MMP-2 promoters inversely correlates with gene expression and cell migration in vitro, with hypomethylated promoters and undermethylated histone H3 being associated with high expression levels of MMP-14 and MMP-2 [172].

MMP-14 expression is also tightly regulated at the transcriptional level. The MMP-14 promoter has at least five different transcription start sites and differs from that of other MMPs in that it does not have a TATA box (Goldberg Hogness box), but has a functional, yet unconventional, binding site for the Sp1 transcription factor [184]. Moreover, binding sites for the activating transcription factors HIF-2α, Egr1, SP1, as well as E2F1, -3 and -5 have been found within the MMP-14 promoter, all of which are associated with increased malignancy in the context of various cancers ([168] and references therein). The collagenous microenvironment of the TME and mechanical forces increase the level of the transcription factor Egr1, resulting in an increased expression of MMP-14 [185,186]. Notably, the collagen receptor α2β1 integrin induces MMP-14 expression in fibroblasts and breast cancer cells [187,188]. Although all 23 human MMP gene promoters have an E2F binding site, only MMP-9, -14, and -15 respond to this transcription factor [189]. Crucially important for the regulation of cancer cell invasion, there is a repressive regulatory PROX1 binding site in front of the main transcription start site [157,184,190].

A decreased expression of the serine/threonine kinase D1 correlates with the invasiveness of breast cancer cells and negatively regulates MMPs -2, -7, -9, -10, -11, -13, -14, and -15 [191,192]. In invasive breast cancer, it is epigenetically shut down by DNA methylation, which induces MMP14 and other MMPs [191].

The synthesis of MMPs is also regulated at the post-transcriptional level by stabilization and destabilization, respectively, of their mRNAs with trans-acting RNA-binding proteins and microRNAs ([9] and references therein).

### 5.5. Regulation of MMP Activity

In addition to MMP activation from inactive zymogen forms, there are diverse other ways of regulating their activity. The proteolytic activity of MMPs as well as ADAMs and ADAMTSs is tightly controlled by the tissue inhibitors of metalloproteinases (TIMPs) that can form 1:1 stoichiometric complexes with MMPs, in which either the N-terminal domain of the TIMP chelates the catalytic zinc ion in the active site of the MMP, thus inactivating it, or in which the C-terminal domain of the TIMP interacts with the hemopexin domain to activate the MMP [193,194,195]. In particular, TIMP-1 is involved in pro-MMP-9/TIMP-1/MMP-3 complex formation to activate MMP-9 [196]. Similarly, TIMP-2 is involved in formation of a proMMP-2/TIMP-2/MMP-14 complex resulting in the activation of proMMP-2 [159]. Two MMP-14 molecules dimerize on the cell surface, whereupon the N-terminal domain of TIMP-2 binds to the catalytic center of one MMP-14, thus facilitating the interaction of the hemopexin domain of proMMP-2 with the C-terminal domain of TIMP-2 to orient the proMMP-2 in such a way that the second MMP-14 can cleave it and release the active MMP-2 [180]. This type of pro-MMP-2 activation occurs particularly in the invadopodia of invading neoplastic cells. Moreover, the homodimerization of two MMP-14 molecules on the cell surface enhances their collagenolytic activity [141]. This is also regulated by interactions with other membrane proteins, such as integrins, CD44, chondroitin/heparin sulfate proteoglycans, and tetraspanins, as well as pericellular MMP-14-inhibiting proteins like TIMP-2, -3, -4, and RECK (reversion-inducing-cysteine-rich protein with Kazal motifs) [141,197]. The invasiveness of cancer cells is likewise regulated by the lifespan of MMP-14, which depends on the pattern of its O-glycosylation, and by the internalization of MMP-14 and the subsequent degradation or recycling to the cell surface [141,198].

In order to enable focalized pericellular ECM proteolysis typical of invading cancer cells, integrin-mediated contacts with the collagen-rich ECM of the tumor stroma bring about a directed transport and fusion of MMP-14-containing vesicles in invadopodia and invasive cell fronts [19,140,143,197]. In particular, caveolin-1 negatively regulates MMP-14 on the cell surface by promoting its internalization [177]. Endocytosed by a flotillin- and Rab5-dependent mechanism and safely stored in intracellular vesicles, such as endolysosomes, activated MMP-14 can be quickly deployed to the cell surface when needed [199,200,201]. The endoplasmic reticulum protein protrudin mediates contact with endosomes containing MMP-14 and the Rab7-binding kinesin adapter protein FYCO1 and facilitates the translocation of MMP-14-containing endosomes to the plasma membrane [202]. The endosomal trafficking of MMP-14 is furthermore regulated by the chloride intracellular channel 4 (CLIC4) which not only binds to the endosomal sorting complex required for transport (ESCRT) but also to proMMP-14 promoting its proteolytic activation in lipid rafts [179].

It has recently been shown that β1 integrin-mediated Src-EGFR signaling regulates MMP-14 phosphorylation and thus the recycling of MMP-14 to sites of invadopodia formation during the invasion of cancer cells [181]. However, it is not yet fully understood, how collagen-binding integrins regulate the expression and activity of MMP-14, and how the topography and biophysical properties of supramolecular collagen affects MMP-14-mediated cancer cell invasion. In fibroblasts migrating through dense collagen, MMP-14 is apparently activated by associating with collagen-binding β1 integrins, especially α2β1, which bring collagen to MMP-14 for proteolytic cleavage [19,197,203]. By this association, proteolytically active MMP-14 may also be kept longer on the cell surface [143]. On the other hand, adhesion- and integrin-mediated signaling via focal adhesion kinase (FAK) and Src can phosphorylate the cytoplasmic tail of MMP-14 and caveolin-1, thereby triggering clathrin- and caveolin-dependent internalization and recycling of MMP-14 [140].

Autocatalytic and non-autocatalytic shedding of the MMP-14 ectodomain from the cell surface yielding either catalytically inactive fragments or soluble catalytically active ectodomains also seems to be a way to regulate its activity and may be important in cancer progression [182,183,204].

MMPs have also been found in mitochondria and in the cell nucleus ([9] and references therein). In hepatocellular carcinoma in particular, MMPs-2 and -14 have been detected inside cell nuclei, although their role there is still unclear [205,206].

### 5.6. Invasive Cancer Cells Breach ECM Barriers with Invadopodia as Drill Heads

Cancer cells form various adhesome structures to interact with their surrounding matrix. Of particular interest for metastasis are special adhesomes termed invadopodia which, in addition to adhesion-promoting integrins, have matrix-degrading enzymes at their proteolytic tip [28,207,208]. In addition to the basement membrane, the dense arrangement of fibrillar collagens, as in the fibrous capsules of desmoplastic tumors, represents an obstacle to the spread of tumor cells (Figure 2) [209]. Integrins, the expression of which is altered in many tumors, play a key role as the most important ECM receptors in the formation of invadosomes. [207,210]. In particular, collagen-binding β1 integrins, but also β3 integrins are involved [181,211]. When invasive cancer cells encounter such rigid collagen fibrils, they break them down similarly to basement membranes using invadopodia [143,212]. MMP-14 resides in such invadopodia and is essential for breaching the ECM barrier and invading the stroma [137,140,159,207]. Invadopodia are spatially complex structures formed in cancer cells under the influence of the TME, whereby in addition to TGF-β, HGF and epidermal growth factor (EGF), hypoxia in particular stimulates their formation [140,213,214,215,216]. Here, the receptors LPA_1_R and EGFR cooperate to promote invadopodia formation via Src and PI3K/Akt signaling [217]. Likewise, via EGFR, the prostaglandin PGE2 receptor EP4 promotes the invadopodia-mediated ECM degradation [218]. Conversely, the tumor suppressor nischarin that interacts with multiple signaling molecules, such as Rac1, LIM kinase (LIMK), liver kinase B1 (LKB1), p21 protein (Cdc42/Rac)-activated kinase 1 (PAK1), Rab14 and insulin receptor substrates 1-4, inhibits the formation of invadopodia and the expression of the integrin subunit α2, while it upregulates subunits α1, α4, and α7 [219]. The strongest stimulus for invadopodia formation is, however, the mechanical stiffness of the ECM, and high collagen density stimulates the incorporation of MMP-14 into tumor cell invadopodia [140,220]. It is sensed by integrins in a Rho- and WASP/WAVE-dependent manner and results in a Rac-, PAK1- and cortactin-dependent formation of the invadopodia’s core structure [94,140,214,221,222,223,224]. At moderate ECM stiffness, cells show rapid invadopodia protrusion–retraction cycles with maximum invadopodia-mediated ECM degradation [225]. The dynamics of invadopodia can also be controlled by the endothelin A receptor ET_A_R via RhoC and cofilin with the help of the scaffold protein β-arrestin1 as a signal-integrating module [216]. F-actin in the core structure is bundled by actinin-1 and -4, with actinin-4 in particular promoting the formation of invadopodia [226]. This core structure of F-actin bundles is further stabilized by recruited signaling molecules such as PAK-1 and -4, thus increasing both the lifespan of the invadopodium and its depth of penetration into the ECM [140,214,227,228]. Other scaffolding proteins stabilize the core structure. FBP17 from the family of F-BAR proteins, which regulate membrane dynamics, is associated with actin-regulatory proteins, such as cortactin, dynamin and ARP2/3 [229]. Similar to cortactin, coronin 1C is required for the formation of invadopodia, but in contrast, it is also involved in the regulation of intracellular endolysosomal transport and the distribution of MMP-14 [230]. By additionally recruiting Tks-4 and -5, invadopodia remain stable for more than 60 min, which distinguishes them from otherwise structurally and functionally similar podosomes of angiogenic ECs that sprout into the tumor mass during tumor-induced angiogenesis [231,232,233]. Invadopodia and podosomes, collectively called invadosomes, both have the proteolytic activity of MMP-14, which enables penetration into the ECM and also activates the soluble gelatinase MMP-2 [140]. In addition to MMP-14, cancer cells have proteinases of the ADAMTS family, which are structurally homologous and likewise membrane-anchored, with which they also can degrade cell migration barriers [234]. Simulations with multiple invadopodia suggest that the distance between individual invadopodia and the secretion rate of soluble MMPs together determine the extent of the ECM degradation. Hence, for invasion cancer cells must fine-tune the inter-invadopodia distance and the secretion rate of soluble MMPs corresponding to the density of the ECM [235].

### 5.7. ECM Degradation and Remodeling Releases Bioactive Matrikines

MMPs are not only essential for ECM remodeling by cleaving structural macromolecules, but they also regulate the release and activation of growth factors, chemokines, cytokines, and adhesion molecules [156]. Furthermore, MMPs may affect the redox conditions in the tumor microenvironment and vice versa, as, for example, MMP-2 and MMP-9 are upregulated by NOX/ROS-dependent NFκB activity [236,237], whereas MMP-3 activation induces mitochondrial ROS production and NADPH oxidase 1 (Nox1) [238]. While the loss of contact with the ECM changes the cellular redox equilibrium leading to apoptosis, cancer cells can recalibrate their redox equilibrium in order to survive and metastasize [239]. Associated with ECM degradation, MMPs proteolytically release from many insoluble structural ECM components soluble bioactive fragments with anti-metastatic properties, referred to as matrikines [240,241,242] (Table 1). Oddly enough, there can be a large number of circulating tumor cells while only one to a few metastases develop, which is due to the release of such antimetastatically acting matrikines produced by proteolytic processing of ECM components in the TME [28,58,243].

The entire TME is significantly influenced by such matrikines which are released by various proteases from insoluble ECM molecules (Table 1). For example, defined fragments of basement membrane collagen types IV, XV, XVIII and XIX, which are split off by infiltrating cancer cells [295], act on the one hand on the cancer cells, and on the other hand, have an angiostatic effect by reducing the sprouting of ECs into the tumor mass [50,240,276,286]. In addition, endostatin can reverse the immunosuppressive environment [102,404], and versicine, a matrikine derived from versican, causes the selective recruitment of certain dendritic cells into the tumor stroma [326]. Furthermore, endorepellin, a fragment of the basement membrane proteoglycan perlecan, can inhibit angiogenesis by interaction with integrin α2β1 on ECs [85,86]. On the other hand, it has been reported that fragments of several matricellular proteins and laminin-332 promote the motility of cancer cells by binding agonistically to the EGF receptor [241,302]. In addition, elastin peptides also act as matrikines and show a broad spectrum of biological activities [300,301,405].

### 5.8. MMPs Promote Epithelial–Mesenchymal Transition

Signals generated by ECM remodeling and degradation play a crucial role in the EMT process during tumor progression by causing numerous structural and functional changes, such as loss of cell polarity and tight intercellular contacts, the production of mesenchymal proteins, and acquisition of an invasive phenotype [406]. In addition to releasing signal-triggering matrikines and breaking ECM barriers, MMPs can proteolytically cleave members of the protease-activated receptor (PAR) family. In particular, the extracellular N-terminus of PARs, such as PAR-1 and PAR-3, which are expressed by cancer cells and also CAFs, can be canonically cleaved by thrombin and also non-canonically by certain MMPs, such as MMP-1 and MMP-13 [407,408,409]. Canonically, thrombin is secreted by activated monocytes/macrophages in the tumor stroma and activated by the extrinsic coagulation cascade that is triggered by the tissue factor (TF) that is usually expressed on cancer cells [410]. Non-canonically, MMPs proteolytically activate the Gα12/13 of the heterotrimeric G protein and thus Rho signaling. This Rho signaling increases cell contractility and cell movement via the actomyosin machinery and its motor protein myosin II, thus stimulating invasion through the ECM barrier. In addition, activated Rho promotes the EMT of cancer cells that are still connected to one another via cadherins. Cadherins can also be cleaved by MMP-14 [164,407,408,411]. Carcinoma cells that invade as coherent cohorts rather than individual cells are interconnected by homophilically interacting E-cadherin [161]. Its loss through cleavage by MMP-14 leads to their spread and changes in their cell morphology, which are characteristic of EMT [164,412].

## 6. Translational Assessment and Future Prospect

To monitor tumor progression and staging, the diagnostic palpatory examination of the correlating tissue stiffening can be refined microscopically histologically and with other imaging methods, such as magnetic resonance imaging (MRI), positron emission tomography (PET), single photon emission computer tomography (SPECT), computed tomography (CT); and ultrasound (US) [413]. For example, changes typical of tumor stroma, such as the appearance of the fibronectin splice variants ED-A or ED-B, laminin-332, periostin and tenascin-W, can be histochemically analyzed in tissue biopsies ([28] and references therein). This allows a prognosis to be made and suggests that such tumor markers may be used for drug development. The small leucine-rich proteoglycan lumican, for instance, inhibits and even reverses some of the characteristics of EMT, such as invadopodia formation, suggesting lumican as a marker for staging of several cancers and the development of cancer drugs based on lumican [414,415]. However, the use of such ECM components as antigenic targets to direct anti-tumor agents to the tumor site has not yet been successful beyond the experimental level, and therefore therapeutics that directly target ECM structural components have to be awaited [416,417].

In addition to structural TME components, ECM receptors can be envisioned as target structures for the pharmacological blocking of tumor progression and metastasis [418,419,420]. Moreover, the inhibitors of MMP-activated PARs that increase metastatic invasion of cancer cells have been investigated in clinical studies [407].

ECM-derived peptides, which are released by enzymes of the TME, are also important not only for diagnostic, prognostic and predictive purposes and as possible therapeutic target structures, but also as lead structures for potential antimetastatic drugs. Their use as antimetastatic therapeutics has been examined in detail in a number of excellent reviews [58,83,85,243,307,419,421,422,423,424,425,426]. Since some of the typical tumor stroma proteins and fragments thereof can be detected diagnostically in blood samples, such as laminin-γ2 chains [89,359], easily accessible and reliable diagnostic, prognostic, and predictive tumor markers may be developed from them.

In addition to structural TME components, the enzyme activities within the TME also represent potential approaches for cancer therapy. Pharmacologically targeting ECM-modifying enzymes such as lysyl oxidase-like LOXLs and MMPs was more successful to date, and some strategies even made it to the stage of clinical trials [129,131,144,427]. In a phase II study, lysyl oxidase activity, involved in forming a premetastatic niche in triple-negative breast cancer, was successfully targeted with copper-scavenging tetrathiomolybdate [428,429]. However, then, simtuzumab, a function-blocking antibody against LOXL2 with an antidesmoplastic effect in vitro, did not improve the clinical results in patients with KRAS-mutated colorectal or pancreatic adenocarcinoma [430,431,432,433].

In recent years, not only the implications of MMPs in cancer progression but also novel strategies for improved targeting and delivery as well as for regulation of MMP activity in tumors have been extensively reviewed [425,434,435]. Inhibitors of ECM-modifying enzymes generally have only a limited selectivity and accordingly show undesirable side effects. Notably, therapeutic strategies for MMP targeting are hampered by limited selectivity. For example, the broad-spectrum MMP inhibitor marimastat, although more bioavailable than its analogue batimastat, turned out unsuitable in phase III studies, since it caused pain and inflammation of the musculoskeletal system [436,437]. Moreover, the development of the low molecular weight inhibitor CGS 27023A/MMI270 targeting MMPs -2, -8, and -9 was stopped early in phase II studies for the treatment of non-small cell lung carcinoma because of poorly tolerated joint and muscle pain [438]. Several new MMP inhibitors with improved properties are currently being investigated ([425] and references therein).

Targeting MMPs therapeutically is obvious, as they are significantly involved in all stages of the metastatic cascade. In particular, inhibiting cancer cell-derived MMPs could increase the stability of the endogenous ECM barrier and thus prevent cancer cells from breaking the basement membrane, which in turn would reduce or even suppress metastases [439,440]. In order to precisely identify tumor tissue during tumor surgery, an MMP-cleavable FRET probe that is internalized by cancer cells has been developed [441]. Elevated serum levels of soluble MMP-14 are a marker for poor prognosis and possibly indicate the presence of distant metastases [442]. In addition, the inhibition of MMP-14 as master MMP is interesting from a therapeutic point of view. A monoclonal antibody against MMP-14 selectively blocks proMMP-2 activation which is required for lymphangiogenesis in vitro and ex vivo [443]. In preclinical models, several monoclonal antibodies against MMP-9 [444,445] and MMP-14 [446] initially appeared promising, but the generation of highly selective MMP antibody inhibitors remains a desideratum [447]. Phage display library-derived peptides against the MT loop of MMP-14, such as HS7, which may be useful in the early diagnosis of tumors and for the development of peptide-mediated drugs [448]. Using a yeast display approach, the rather non-specific MMP inhibitor N-TIMP-2 could be converted into a specific inhibitor that blocks MMP-9 activity thousand-fold more than MMP-14 activity [449].

In view of the recently enormously growing understanding of gene regulation in TME, regulatory RNAs appear attractive, since diagnostic, prognostic and predictive biomarkers could be found in both microRNAs and long non-coding RNAs [450,451]. In particular, microRNA-mediated post-transcriptional MMP regulation is of interest for tumor therapy [452]. For example, miR-181a-5p can downregulate MMP-14 and thereby inhibit the migration and angiogenesis of cancer cells [453]. Similarly, miR139-5p and miR203 slow down processes involving the gelatinase MMP-9 and the matrilysin MMP-7, respectively [454,455]. Other microRNAs, such as miR192 and miR140-5p are involved in the upregulation of MMP-2 [456] and ADAMTS5 [457], respectively, suggesting the development and use of respective antagomiRs. It is also conceivable to inhibit oncomiRs, e.g., miR-21 in order to upregulate the expression of MMP-regulating proteins, such as RECK or TIMP-3, to reduce MMP activity [458].

Whatever the methodological approach, attempts to normalize the dysregulated TME and its ECM in a non-tumor-supporting environment [459] and to prevent the tumor-induced formation of premetastatic niches [460,461] are, in any case, goals of prime importance.

## Figures and Tables

**Table 1:** Matrix proteins of the TME can release bioactive peptides called matrikines, which regulate tumor progression and metastasis, and can be used diagnostically.

Matrix Protein	BM Component	Protease	Bioactive Peptide Released	Receptor	Potential Use in Diagnostics
**Collagens** [5,16,244]					
**Fibrillar collagens**					
Procollagen I		BMP-I	P1CP [245,246,247]		
Procollagen IIB		ADAMTS-3 [248]	Chondrostatin [249,250]	Integrins αvβ3, αvβ5 [249]	
Procollagen III			PRO-C3 [251,252]		Associated with shorter TTP and OS in metastatic breast cancer [251]
Collagen I		Collagenases MMPs -1, -8, -14		Integrins α1β1, α2β1, α10β1, α11β1, DDR1, DDR2, GPVI, LAIR-1, MR, PLA2R, LY75, Endo180 ([253] and references therein)	
		MMPs -2, -9, -13 [251]	C1M [251,254]		Associated with shorter TTP and OS in metastatic breast cancer [251]
			PGP (pro–gly–pro) [255,256]	CXCR2	
Collagen II		Gelatinases MMPs -2, -9 [135,138,145]			
Collagen III [257]		MMP-9 [251]	C3M [251,258]		Associated with shorter TTP and OS in metastatic breast cancer [251]
Collagen V [257]					
Collagen XI [257]					
Collagen VI		MMP-11	α3(VI) endotrophin [259,260,261]		
Collagen VIII			α1(VIII) vastatin [262,263,264,265]		Correlates with ECM degradation and stromal reactivity; increased in serum in colorectal cancer [262]
**Network-forming collagens and multiplexins** (e.g., in BMs) [266,267,268]	Yes	MT-MMPs [269], cathepsin S [270], MMPs -2, -3, 9, -13			
Collagen IV		Cathepsin S [270], MMP-14, 15 [269], MMP-9 [271]	α1(IV) arresten [272], α2(IV) canstatin [273], α3(IV) tumstatin [271,274], α4(IV) tetrastatin [275,276], α5(IV) pentastatin [275], α5(IV)NC1 lamstatin [277], α6(IV) hexastatin [275]	Integrins α1β1, α2β1, αVβ3, αvβ5 bind arresten, canstatin, tumstatin [85,240,278,279]	
			α6(IV)NC1 [280]	Iintegrin αVβ3 [281]	
		MMPs -9, -12 [251]	C4M [251,282]		Associated with shorter TTP and OS in metastatic breast cancer [251]
Collagen XV			NC α1(XV) restin		
Collagen XVIII		MMPs -3, -9, -12, -13, -20 [283], cathepsins L, S [284], elastase [285]	α1 (XVIII) endostatin [85,240,276,283,286]	Nucleolin [287], integrins α5β1, αVβ3, αvβ5 [85], caveolin-1 [288], VEGFR2 [289], glypican-1, 2 [290]	
		MMP-7 [291]	Neostatin 7 [291]		
		MMP-14 [292]	Neostatin 14 [292]		
**FACITs** (fibril-associated collagens with interrupted triple helix)					
Collagen IX [293]					
Collagen XIX		Plasmin [294]	NC-1 α1(XIX) [295]	Integrin αvβ3 [296]	
**Collagen of anchoring fibrils**	Yes				
Collagen VII		MMP-1 [297]	NC1		
**Elastic fibrils**					
Elastin [298,299]		Ela-2, cathepsin G, proteinase-2, cathepsins L, S, K, V, MMPs -2, -7, -9, 12 [300] MMPs -1, -2, -8, -9, -12 [125]	Elastin-derived matrikine, VG-6 (VGVAPG), AG-9 (AGVPGLGVG) [125,241,301,302]	Elastin receptor complex (ERC) [300]	
Fibrillin [298]		ADAMTS -10, -6 [303]		Integrins αVβ3, αVβ6, α5β1 [304,305]	
**Fibronectins** [29,306,307,308,309]	Yes	MMPs -2, -3, -7, -10, -11 [297]	Fibronectin fragments (FNFr) [310], anastellin [311], fibstatin [312]	Integrins α5β1 [313], α9β1, α4β1, αv-integrins [309,314,315], growth factors and syndecans [316]	
Fibronectin ED-A [29]				Integrins α9β1, α4β1, α5β1, αv-integrins [29,308,317]	Marks tumor stroma [29,306,307,308,315,317,318,319,320]
Fibronectin ED-B [29]				Integrins α9β1, α4β1, α5β1, αv-integrins [29,308,317]	Marks tumor stroma [29,306,307,308,315,317,318,319,320]
**Proteoglycans**					
**Hyalectans (Lecticans)**					
Aggrecan		Aggrecanases, MMPs -1, -2, -3, 7, -8, -9, -13, -14 ([321] and references therein), ADAMTSs [234,321]			
Neurocan				NCAM-L1, indirectly N-cadherin [322] and NrCAM/Sema3F [323]	
Brevican		ADAMTSs [324]			
Versican		ADAMTS -4, -5 [325]	Versican-derived matrikine, versicine [326,327]	β1 integrins [328]	
**SLRPs** [4,83,84]					
Decorin		MMPs -2, -3 [329]		EGFR [330], IGF-1R ([331] and references therein), MET [332], VEGFR2 [333], 51 kD receptor [334]	
Biglycan				TLR-2, TLR-4, LRP6, MuSK ([335] and references therein), 51 kD receptor [334]	
**Perlecan**	Yes	MMP-3, -7 [329,336], cathepsin L [337]	Endorepellin [85,86]	VEGFR2/integrin α2β1 [86,338,339]	Blood levels of domain IV fragments elevated in prostate carcinoma [336]
		BMP1/TLD-like protease [340], cathepsin L, t-PA [337]	LG3 fragment (C-terminal fragment of endorepellin)		
**Glypican-3**			glypican-3 derived peptide [340]		
**Nidogen-1**	Yes	MMP-19, cathepsin S, meprin A [341,342,343]	G3 domain		
**Laminins** [21,23,344]	Yes	MMPs -2, -3, -7, -10 [297,329]			Mark BMs
α chain			α1 chain: IKVAV, RKRLQVQLSIRT (AG-73) [345]	Integrins α3β1, α6β1, syndecans 1, 2, 4 [345]	
			α3 chain: C-terminal fragment		
			α5 chain: AQARSAASKVKVSMKF [346]	Heparan sulfate proteoglycans [50],syndecans [347,348,349]	
β chain			β1 chain: YIGSR [345]	67 kD receptor [345]	
γ chain			γ1 chain: KAFDITYVRLKF (C16) [345]	Integrins αvβ3, α5β1 [345]	
Laminin-332	Yes	MMPs -2, -9 [241,297,329]	LG3, LG4 [350,351,352,353], EGF-L repeats ([241] and references therein), γ2 chain: N-terminal fragment	Integrin α3β1 [354], EGFR ([241] and references therein)	Marks tumor stroma [352,355,356]; β3 marks tumor stroma, poor prognosis [357,358], γ 2 marks tumor stroma, poor prognosis, γ2 in blood samples [358,359]
Laminin-511			RLVSYNGIIFFLK (A5G27) [360,361]		
**Matricellular proteins**					
CCNs [44]:					
CCN1 (CYR61)				Integrins αvβ3, αvβ5, α6β1, syndecan-4 [362,363,364]	
CCN2 (CTGF)			CCN2-fragments [365]	Integrin α6β1, αvβ3 [366,367]	Marks vasculogenic mimicry [368,369,370,371]
Tenascins [372,373]					
Tenascin C [374]		MMPs -1, -8, -13 [329]	EGF-L repeat [241]	Integrin α9β1 [37], EGFR (EGF-L) ([241] and references therein)	
Tenascin W [375]					Marks tumor stroma [35,375,376,377]
Thrombospondins [298]				CD36, αV and β1 integrins, syndecan, CD47	
Osteopontin [378,379,380]					Marks tumor progression [381]
Periostin [382]				Integrins αVβ3, αVβ5 [383]	Marks tumor stroma [40,358,382,384,385,386,387,388,389,390]
SPARC [391]					Abundant in healthy vessels and tumors of good prognosis [391]
Galectins [392]					Promote tumor angiogenesis [393] and affect tumor immunology [394]
**SIBLINGs** [44,395]					
Bone sialoprotein					Marks tumor progression [381]
Dentin matrix protein I					
Sialophosphoprotein					
Matrix extracellular glycoprotein					
Syndecans [396]					
Syndecan-1			Synstatins SSTN_92-119_ [397,398,399], SSTN_82-130_ [400], SSTN_210-240_ [399,401]		
Syndecan-4			SSTN_87-131_ [399]		
**Agrin**		neurotrypsin [402]	C-terminal agrin fragment [402]		Not yet found related to the tumor microenvironment
**Hyaluronan** [53]					
Hyaluronic acid		HYAL2 [73,403]	HA oligosaccharides [127]	CD44, RHAMM, TLR4 [75]	

Various bioactive peptides that can be released by proteolytic cleavage from the ECM of the TME are of interest for diagnosis. These peptides elicit different cell functions through their receptors. Please refer to the text for further information. Updated from [28]. Abbreviations: BM, basement membrane; G3 domain, globular 3 domain; Endorepellin LG3 domain, Endorepellin laminin-like globular 3 domain; HYAL2, hyaluronidase 2; MMP, matrix metalloproteinase; SLRP, small leucine-rich protein ; VEGFR2, vascular endothelial growth factor tyrosine kinase receptor 2; t-PA, tissue-type plasminogen activator; BMP1/TLD-like protease, bone morphogenetic protein 1/tolloid-like protease; CSPGs, chondroitin sulfate proteoglycans; OS, overall survival; TLR2, Toll-like receptor; TTP, time to progression.

## Abbreviations

ADAM(TS)	a disintegrin and a metalloproteinase (with thrombospondin-1 motif)
BM	basement membrane
CAF	cancer-associated fibroblast
CAM	cell adhesion molecule
CCN	connective tissue growth factor (CTGF), cysteine-rich protein (Cyr61) and nephroblastoma overexpressed (NOV)
EC	endothelial cell
ECM	extracellular matrix
ED-A/B	extra domain-A/B
EGF(R)	epidermal growth factor (receptor)
EMT	epithelial–mesenchymal transition
GAG	glycosaminoglycan
GPI	glycosylphosphatidylinositol
HGF	hepatocyte growth factor
HIF	hypoxia-inducible factor
H/LMW-HA	high/low molecular weight hyaluronic acid
LOXL	lysyl oxidase-like
LPA_1_R	lysophosphatidic acid receptor 1
(MT-)MMP	(membrane-type) matrix metalloproteinase
NOX	nicotinamide adenine dinucleotide phosphate (NADPH) oxidase
PAR	protease-activated receptor
RHAMM	receptor for hyaluronic acid-mediated migration
ROS	reactive oxygen species
SIBLING	small integrin-binding ligand N-linked glycoprotein
SLRP	small leucine-rich protein
SPARC	secreted protein acidic and rich in cysteine
TC	tumor cell
TGF-β	transforming growth factor- β
TIMP	tissue inhibitors of metalloproteinases
TLR	Toll-like receptor
TME	tumor microenvironment
VEGF	vascular endothelial cell growth factor
